# Nicotinamide riboside as a neuroprotective therapy for glaucoma: study protocol for a randomized, double-blind, placebo-control trial

**DOI:** 10.1186/s13063-021-05968-1

**Published:** 2022-01-17

**Authors:** Christopher Kai Shun Leung, Seraph Tianmin Ren, Poemen Pui Man Chan, Kelvin Ho Nam Wan, Aziz Ka Wai Kam, Gilda Wing Ki Lai, Vivian Sheung Man Chiu, Match Wai Lun Ko, Cedric Ka Fai Yiu, Marco Chak Yan Yu

**Affiliations:** 1 Department of Ophthalmology, LKS Faculty of Medicine, The University of Hong Kong, Hong Kong, People’s Republic of China; 2 Hong Kong Eye Hospital, Hong Kong, People’s Republic of China; 3 Department of Ophthalmology and Visual Sciences, The Chinese University of Hong Kong, Hong Kong, People’s Republic of China; 4 Department of Mechanical Engineering, University of Hong Kong, Hong Kong, People’s Republic of China; 5 Department of Applied Mathematics, Hong Kong Polytechnic University, Hong Kong, People’s Republic of China; 6 Singapore Eye Research Institute, Singapore, Singapore

**Keywords:** Glaucoma, Nicotinamide riboside, Retinal nerve fiber layer, Progressive retinal nerve fiber layer thinning, Optical coherence tomography, Visual field, Neuroprotection

## Abstract

**Background:**

Whereas lowering the intraocular pressure (IOP) can slow optic nerve degeneration in glaucoma, many patients with glaucoma continue to develop progressive loss in vision despite a significant reduction in IOP. No treatment has been shown to be effective for neuroprotection in glaucoma. We set out to conduct a randomized controlled trial to investigate whether nicotinamide riboside (NR), a nicotinamide adenine dinucleotide precursor, is effective to slow optic nerve degeneration in patients with primary open-angle glaucoma (POAG). We hypothesize that patients treated with NR have a slower rate of progressive retinal nerve fiber layer (RNFL) thinning compared with those treated with placebo.

**Methods:**

This is a randomized, double-blind, placebo-controlled, parallel-group, multi-center study including 125 patients with POAG. Patients will be randomized to receive 300 mg NR or placebo for 24 months. Clinical examination, optical coherence tomography imaging of the RNFL, and visual field (VF) test will be performed at the baseline, 1 month, 4 months, and then at 2-month intervals until 24 months. The primary outcome measure is the rate of RNFL thinning measured over 24 months. The secondary outcome measures include (1) time to VF progression, (2) time to progressive RNFL/ganglion cell inner plexiform layer (GCIPL) thinning, and (3) the rate of change of VF sensitivity over 24 months (to investigate neuroprotection) and 1 month (to investigate neuroenhancement). The rates of RNFL thinning and VF sensitivity decline between treatment groups will be compared with linear mixed modeling. Survival analysis will be performed to compare the differences in time from baseline to VF progression and time from baseline to progressive RNFL/GCIPL thinning between treatment groups using Cox proportional hazards models.

**Discussion:**

Outcome measures in glaucoma neuroprotection trials have been centered on the detection of VF progression, which may take years to develop and confirm. In addition to addressing whether NR has a neuroprotective/neuroenhancement effect in glaucoma patients, this study will demonstrate the feasibility of studying neuroprotection in a relatively short trial period (24 months) by comparing the rates of progressive RNFL thinning, a more reproducible and objective outcome measure compared with VF endpoints, between treatment groups.

**Trial registration:**

Chinese Clinical Trial Registry 1900021998

## Administrative information

Note: the numbers in curly brackets in this protocol refer to SPIRIT checklist item numbers. The order of the items has been modified to group similar items (see http://www.equator-network.org/reporting-guidelines/spirit-2013-statement-defining-standard-protocol-items-for-clinical-trials/).

**Table:** 

Title {1}	Nicotinamide Riboside as a Neuroprotective Therapy for Glaucoma: Study Protocol for a Randomized, Double-blind, Placebo-control Trial
Trial registration {2a and 2b}.	Chinese Clinical Trial Registry 1900021998 Date of registration: 21 October 2019 http://www.chictr.org.cn/historyversionpuben.aspx?regno=ChiCTR1900021998 Chinese Clinical Trial Registry is in the WHO Registry Network and meets the requirements of the ICMJE.
Protocol version {3}	Version 5 (dated 8 Nov 2020)
Funding {4}	Hong Kong Food and Health Bureau, Health and Medical Research Fund 06171076; nicotinamide riboside is sponsored by the Li Ka Shing Foundation Limited
Author details {5a}	Department of Ophthalmology, The University of Hong Kong
Name and contact information for the trial sponsor {5b}	Food and Health Bureau Research Fund Secretariat: enquiry@fhb.gov.hk Li Ka Shing Foundation Limited: general@lksf.org
Role of sponsor {5c}	The study sponsor and funder have no roles in study design; collection, management, analysis, and interpretation of data; writing of the report; and the decision to submit the report for publication. They do not have ultimate authority over any of these activities.

## Introduction

### Background and rationale {6a}

Glaucoma, characterized by progressive degeneration of the optic nerve or the axons of retinal ganglion cells, is the leading cause of irreversible blindness worldwide with 76 million patients worldwide in 2020 [1, 2]. Although lowering the intraocular pressure (IOP) can decrease the rate of visual field (VF) sensitivity worsening, many patients with glaucoma continue to show a progressive loss in vision despite a significant reduction in IOP [3, 4]. The need to investigate treatment to protect the optic nerve other than IOP reduction is pressing. At present, there is no neuroprotective therapy approved for the treatment of glaucoma.

Nicotinamide riboside (NR) is a precursor of nicotinamide adenine dinucleotide (NAD), which is a key cofactor in redox metabolism including glycolysis, citric acid cycle, and oxidation phosphorylation as well as in non-redox cell signaling and gene regulation [5–7]. Increasing evidence has shown that axon degeneration is associated with a decline in NAD and that supplementation of NAD or NAD precursors is effective to delay axon degeneration in vitro and in vivo [7–10]. In an experimental study, Williams and colleagues demonstrated oral administration of nicotinamide was able to increase the levels of NAD in the retina and protect retinal ganglion cells from degeneration in a mouse model of glaucoma [11]. Remarkably, 93% of eyes did not develop glaucomatous retinal ganglion cell loss despite elevated IOP in animals treated with a high dose of oral nicotinamide (2000 mg/kg/day). However, high-dose nicotinamide can induce hepatotoxicity and painful flushing [12]. Nicotinamide riboside, by contrast, is more orally bioavailable than nicotinamide to increase tissue levels of NAD [6]. It is a well-tolerated NAD precursor with a high safety profile [13, 14]. Although oral administration of NAD precursors affords protection against glaucoma and neurodegenerative diseases in animal models, lost in translation to clinical practice is common in neuroprotection studies [15, 16]. We set out to perform a randomized controlled trial to determine whether NR can protect the optic nerve in patients with glaucoma.

## Objectives {7}

The study aims to investigate if oral supplementation of NR is effective to decrease the rate of progressive retinal nerve fiber layer (RNFL) thinning in glaucoma.

## Trial design {8}

**Fig. 1:**
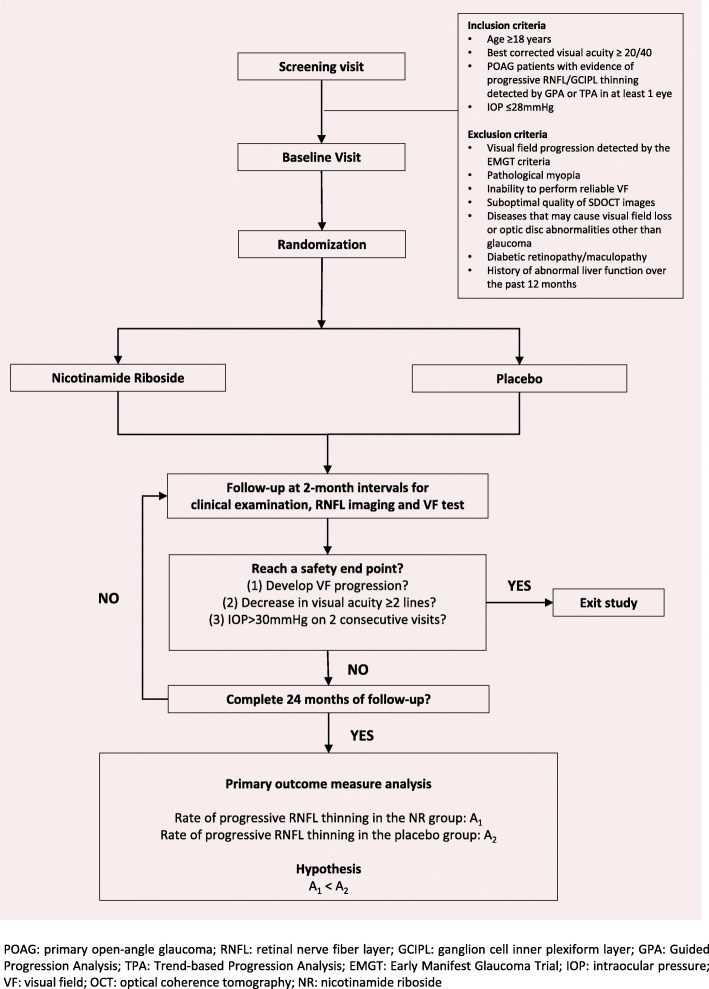
Flow chart of the study design. *POAG* primary open-angle glaucoma, *RNFL* retinal nerve fiber layer, *GCIPL* ganglion cell inner plexiform layer, *GPA* Guided Progression Analysis, *TPA* Trend-based Progression Analysis, *EMGT* Early Manifest Glaucoma Trial, *IOP* intraocular pressure, *VF* visual field, *OCT* optical coherence tomography, *NR* nicotinamide riboside

This study is a double-blind, parallel-group (1:1), randomized, controlled, superiority trial. The trial will randomize 125 patients with primary open-angle glaucoma (POAG) with evidence of progressive RNFL thinning or ganglion cell inner plexiform layer (GCIPL) thinning in optical coherence tomography (OCT) without visual field (VF) progression in at least one eye to receive oral NR 300 mg/day or placebo. Patients will be followed up at 1 month, 4 months, and then every 2 months until 24 months for comparisons of the rates of RNFL thinning and the rates of change of VF sensitivity between treatment groups. The same IOP-lowering regimen as prescribed before study recruitment will be provided throughout the 24-month study period unless a safety endpoint (described below) is reached. Change in VF sensitivity at 1 month will be compared between treatment groups to investigate neuroenhancement. We hypothesize that patients treated with NR have a slower rate of RNFL thinning and a slower rate of VF sensitivity decline compared with those treated with placebo over 24 months and that an improvement in VF sensitivity could be detected in the NR-treated group at 1 month. The study flow chart is shown in Fig. 1.

## Methods: participants, interventions, and outcomes

### Study setting {9}

Patients with primary open-angle glaucoma will be consecutively recruited from Hong Kong Eye Hospital, Chinese University of Hong Kong (CUHK) eye clinic, and Prince of Wales Hospital eye clinic. These patients will be followed up at the CUHK eye clinic and the University of Hong Kong Glaucoma and Optic Nerve Diagnostic Center for clinical examination, OCT imaging and VF testing.

### Eligibility criteria {10}

Inclusion criteria are as follows: age ≥ 18 years, best corrected VA ≥20/40, IOP ≤28 mmHg, and evidence of progressive RNFL/GCIPL thinning detected by Guided Progression Analysis (GPA, Carl Zeiss Meditec) or Trend-based Progression Analysis (TPA) in one or both eyes [17–20]. Exclusion criteria are as follows: VF progression detected by the Early Manifest Glaucoma Trial (EMGT) criteria (likely progression in GPA) [21] over 18 months prior to study enrollment, pathological myopia, diseases that may cause visual field loss or optic disc abnormalities other than glaucoma, inability to perform reliable visual field, suboptimal quality of OCT images, diabetic retinopathy/maculopathy, and a history of abnormal liver function within 12 months.

### Who will take informed consent? {26a}

The principal investigator will take informed consent after explaining the potential benefits and downsides of participation, the details of clinical investigations performed during the study follow-up, and the right to withdraw from the study. Patients will be given at least 1 week to consider the participation and raise any questions at their concern.

### Additional consent provisions for collection and use of participant data and biological specimens {26b}

This trial does not involve collecting biological specimens for storage.

## Interventions

### Explanation for the choice of comparators {6b}

Patients randomized to the control arm will be provided a placebo containing corn starch. The placebo capsule has identical color, appearance, and packaging as the capsule containing NR.

### Intervention description {11a}

During the 24-month study period, patients randomized to the NR treatment group will have oral administration of 3 capsules of NR every morning (each capsule containing 100 mg NR); patients randomized to the controlled group will have oral administration of 3 capsules of placebo every morning. Patients will be followed up at 1 month, 4 months, and then every 2 months for clinical examination, VF testing, and OCT imaging of the RNFL/GCIPL.

### Criteria for discontinuing or modifying allocated interventions {11b}

The safety endpoints are (1) development of VF progression (likely progression in GPA according to the EMGT criteria) [21], (2) decrease in visual acuity ≥2 lines, and (3) IOP > 30 mmHg on 2 consecutive visits. Patients will exit the study and receive appropriate treatment if any of the safety endpoints is reached. Patients are allowed to withdraw from the study for any reasons at any time.

### Strategies to improve adherence to interventions {11c}

The capsules (NR or placebo) are packed in weekly packets (each packet contains 21 capsules—3 capsules per day for 7 days) labeled with patient’s name. Patients are requested to bring back the packets at each follow-up visit. Capsules left in the packets will be counted to check and remind patients’ compliance to the treatment.

### Relevant concomitant care permitted or prohibited during the trial {11d}

Patients will continue the existing IOP-lowering regimen as prescribed before study enrollment. No additional IOP-lowering intervention will be provided unless a safety endpoint is reached (i.e., detection of VF progression, decrease in visual acuity ≥2 lines, or IOP > 30 mmHg on 2 consecutive visits). They are allowed to take other medications for medical conditions during the trial. Details of the medications used during the study period will be recorded and reported.

### Provisions for post-trial care {30}

The no-observed-adverse-effect-level of NR was 300 mg/kg/day [14]. We do not expect to observe any adverse effect with an oral intake of NR at 300 mg/day.

### Outcomes {12}

The primary outcome measure is the rate of change of average RNFL thickness measured by OCT over 24 months of study follow-up. Secondary outcome measures include (1) time to VF progression (possible or likely VF progression in GPA according to the EMGT criteria [21]) over 24 months expressed in survival probability, (2) time to progressive RNFL/GCIPL thinning (defined by Trend-based Progression Analysis [17, 18, 20]) over 24 months expressed in survival probability, and (3) the rate of change of VF sensitivity over 24 months (to investigate neuroprotection) and 1 month (to investigate neuroenhancement). Change in inner retinal vessel density over the parapapillary region and the macula measured by OCT angiography over 24 months will be evaluated as an exploratory outcome measure.

### Participant timeline {13}

**Fig. 2:**
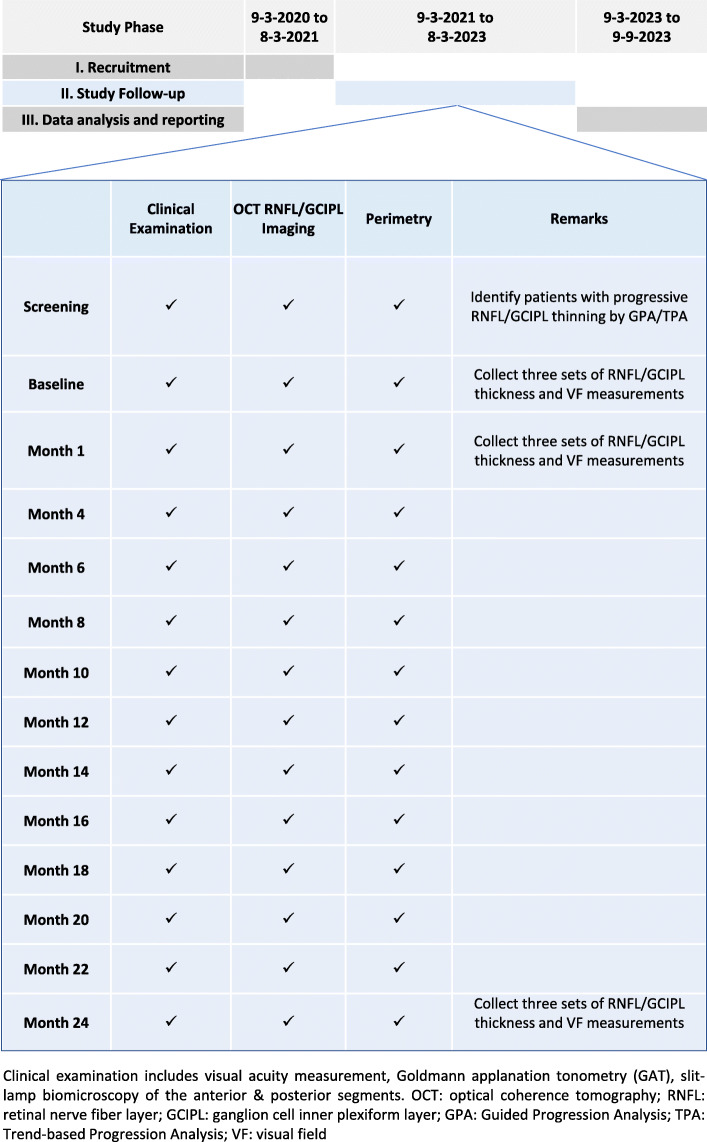
Study timeline. Clinical examination includes visual acuity measurement, Goldmann applanation tonometry (GAT), and slit-lamp biomicroscopy of the anterior and posterior segments. *OCT* optical coherence tomography, *RNFL* retinal nerve fiber layer, *GCIPL* ganglion cell inner plexiform layer, *GPA* Guided Progression Analysis, *TPA* Trend-based Progression Analysis, *VF* visual field

Study enrollment started on 9 March 2020, and the expected completion date of recruitment is 8 March 2021. Patients will be followed up at 1 month, 4 months, and then every 2 months until 24 months for clinical examination, IOP measurement, OCT imaging of the RNFL/GCIPL, and VF testing (Fig. 2).

### Sample size {14}

In our pilot study following 236 glaucoma patients every 4 months for ≥3 years, the mean rate of RNFL thinning was 1.2 μm/year (*P* < 0.001) for eyes with evidence of progressive RNFL thinning detected by TPA; the slope variance was 1.39 (μm/year)^2^ and the residual variance was 3.83 μm^2^ [20]. We aim to detect a difference of at least 0.7 μm/year in the rate of RNFL thinning between treatment groups, given that the mean rate of age-related RNFL thinning has been reported to be approximately 0.5 μm/year [22]. Taking reference from the method of sample size calculation for linear mixed modeling to compare the rates of change of a parameter of interest between two treatment groups described by Ard and Edland [23], 56 patients per study arm are needed for serial RNFL thickness measurements at baseline, 1 month, 4 months, and then every 2 months for 24 months (3 separate measurements will be collected at the baseline, 1-month, and 24-month follow-up visits) with a power of 80% and an alpha of 5%. Assuming a default rate of 10% during study follow-up, a total of 125 patients will be recruited.

### Recruitment {15}

Attending ophthalmologists and investigators will review the clinical records and the RNFL/GCIPL progression analysis reports for glaucoma patients coming back to the eye clinics for scheduled follow-up appointments. Patients with at least 1 year of serial OCT RNFL/GCIPL measurements prior to the recruitment will be checked by the investigators for evidence of progressive RNFL/GCIPL thinning. Those with evidence of progressive RNFL/GCIPL thinning detected by Guided Progression Analysis (GPA) or Trend-based Progression Analysis (TPA) in at least one eye will be invited to attend a screening visit. The principal investigator will explain the study procedures to the patients. Patients who agree to participate in the study will come back on a scheduled visit for baseline investigation.

## Assignment of interventions: allocation

### Sequence generation {16a}

Patients will be randomly allocated (1:1) at the baseline visit to receive NR or placebo within 1 month of enrollment. Randomization with minimization by age (< 60 years vs ≥60 years), gender (male vs female), VF MD (<−6 dB vs ≥ −6 dB), spherical equivalent (<−6.0D vs ≥ −6.0D), mean IOP measurement over the past 3 years (≤21 mmHg vs > 21 mmHg), and number of glaucoma medications (0, 1, 2, 3, or ≥ 4) will be performed using an open-source computer program MinimPy [24]. A random element was introduced in the minimization procedure (i.e., weighted randomization of 0.8) to make the randomization more unpredictable [25]. If both eyes are eligible for inclusion, the one with smaller VF MD will be selected for randomization with minimization.

### Concealment mechanism {16b}

Patients and investigators are unaware of treatment allocations and have no access to the randomization sequence and codes, which are stored in a password-protected file in a computer. The NR and placebo tablets are in identical appearance and uniformly packaged.

### Implementation {16c}

The principal investigator will enroll participants. Two masked research technicians will generate the allocation sequence with randomization by minimization and assign participants to interventions.

## Assignment of interventions: blinding

### Who will be blinded {17a}

The attending ophthalmologists, the participants, the outcome assessors, and data analysts will be blinded after assignment to intervention.

### Procedure for unblinding if needed {17b}

The blinding will be maintained until a serious adverse event occurs. Unblinded participants will exit the trial and the ocular and/or medical conditions will be managed accordingly. The management results will be recorded on the clinical report form and reported to the Hong Kong Hospital Authority Cluster Research Ethics Committee.

## Data collection and management

### Plans for assessment and collection of outcomes {18a}

Patients will receive a clinical examination, OCT imaging of the RNFL, and VF testing at baseline, 1 month, 4 months, and then every 2 months for 24 months. Three repeated OCT and VF measurements will be collected at the baseline, 1-month, and 24-month visits.

#### Clinical examination

Clinical examination includes slit-lamp biomicroscopy of the anterior and posterior segments and Goldmann applanation tonometry. Two IOP readings will be obtained to calculate the mean. A third reading will be obtained if the difference between the first two is > 2 mmHg and the median is recorded. Axial length will be measured with partial coherence laser interferometry (IOLMaster, Carl Zeiss Meditec). Subjective and objective refraction will be performed at the screening and the last follow-up visits. Central corneal thickness will be measured with anterior segment optical coherence tomography (CASIA2, Tomey, Japan). Dilated fundus examination for color optic disc stereophotography will be performed at the screening visit and then yearly.

#### Optical coherence tomography imaging

The RNFL will be imaged with the Cirrus HD-OCT (Carl Zeiss Meditec, Dublin, USA) for comparison of the rates of RNFL thinning (primary outcome measure) between treatment groups. The RNFL-GCIPL will be imaged by the Triton OCT (Topcon, Tokyo, Japan) to determine the time to progressive RNFL-GCIPL thinning detected by TPA for an individual eye (secondary outcome measure). Low test-retest variability of Cirrus HD-OCT and Triton OCT measurements has been shown [26, 27]. RNFL-GCIPL thickness data of individual pixels in serial RNFL-GCIPL thickness maps collected over 24 months will be exported for TPA [17, 18, 20]. Trend-based Progression Analysis performs pixel-by-pixel linear regression analysis between RNFL-GCIPL thickness and time and determines progressive RNFL-GCIPL thinning after registering and aligning serial OCT scans in corresponding retinal locations of an eye. To minimize type I errors consequential to multiple testing in an eye, the RNFL-GCIPL thickness maps will be condensed from 512 × 256 pixels to 128 × 64 superpixels with a false discovery rate (FDR) controlled at 5%. Images with a poor signal-to-noise ratio, motion artifact, poor centration, segmentation error, or missing data (e.g., blinking) will be discarded; re-scanning will be performed in the same visit. Peripapillary and macular inner retinal vessel density will be measured with OCT angiography.

#### Visual field test

The visual field test will be performed with the Humphrey Field Analyzer II 24-2 SITA standard strategy (Carl Zeiss Meditec). A reliable VF test has fixation losses < 20% and false-positive rate < 15%. Unreliable tests will be repeated on the same day. Visual field progression is identified when three test locations show a significant reduction in VF sensitivity greater than the test-retest variability observed on at least two (possible progression) or three (likely progression) consecutive tests, according to the EMGT criteria [21].

### Plans to promote participant retention and complete follow-up {18b}

Investigators will phone contact or send a text message to remind the participants for follow-up before each study visit. Participants who default a scheduled appointment will be contacted by the investigators to re-arrange another appointment within 2 weeks.

### Data management {19}

Clinical data will be collected and recorded by two designated technicians. Clinical examination data (e.g., IOP and axial length) will be entered on case report forms and then entered electronically. Consistency checks by another technician will be performed to ensure data entry accuracy. Optical coherence tomography RNFL/GCIPL thickness and VF sensitivity data be directly exported from the OCT and VF instruments to avoid data entry errors. All data will be stored in password-protected computers. The study will be conducted in compliance with Good Clinical Practices to ensure the rights and well-being of the participants and that the data collected are complete and verifiable from source documents. Patient files will be maintained in storage for a period of 3 years after completion of the study.

### Confidentiality {27}

No personal information will be recorded on the data sheets or electronic data files. A study code will be assigned to each participant. The document containing the information of the study code and the identity of the patient will be kept separate from the study data files and data sheets.

### Plans for collection, laboratory evaluation, and storage of biological specimens for genetic or molecular analysis in this trial/future use {33}

There are no plans for collection of biological specimens in this trial.

## Statistical methods

### Statistical methods for primary and secondary outcomes {20a}

(1) The rates of RNFL thinning (primary outcome measure) between treatment groups will be compared with linear mixed modeling after adjusting for covariates including age, axial length, glaucoma severity (baseline RNFL thickness), IOP levels during following up, OCT signal strength, multiple testing, and clustering between fellow eyes. (2) Change in VF sensitivity (measured in dB) at 1 month and the rate of change in VF sensitivity over 24 months between treatment groups will be compared with linear mixed models after adjusting for covariates. (3) Survival analysis will be performed to compare the differences in survival probabilities in (A) time from baseline to VF progression (determined by the EMGT criteria) and (B) time from baseline to progressive RNFL-GCIPL thinning (determined by TPA) between treatment groups using Cox proportional hazards models. One eye from each patient (the one selected for randomization with minimization) will be included in the analyses.

### Interim analyses {21b}

Interim analyses will be performed by a statistician after data collection at (A) 4 months and (B) 12 months. The rates of change of VF sensitivity and RNFL thickness will be compared between treatment groups. The participants and investigators will remain masked to the study groups. The trial steering committee will stop the trial if the rate of VF sensitivity decline and the rate of RNFL thinning were significantly worse in the NR-treated group than the placebo-treated group.

### Methods for additional analyses (e.g., subgroup analyses) {20b}

Detection of progressive RNFL thinning and VF sensitivity decline has been shown to be more difficult in moderate to advanced glaucoma compared with early glaucoma. Subgroup analysis will be performed to determine if the rates of change of RNFL thickness and VF sensitivity would be different between treatment groups in patients with early glaucoma versus those with moderate to advanced glaucoma. Subgroup analysis will also be performed to investigate if the responses of NR supplementation would be different between old and young patients.

### Methods in analysis to handle protocol non-adherence and any statistical methods to handle missing data {20c}

Data will be analyzed on an intention-to-treat basis to include all patients who receive the randomized treatment and have at least two follow-up visits. Multiple imputation by chained equations (MICE) algorithm will be applied for estimation of missing data [28].

### Plans to give access to the full protocol, participant-level data, and statistical code {31c}

No later than 3 years after the completion of data collection, the protocol and deidentified dataset will be available from the principal investigator upon reasonable request.

## Oversight and monitoring

### Composition of the coordinating center and trial steering committee {5d}

The coordinating center is located at the University of Hong Kong Glaucoma and Optic Nerve Diagnostic Center. The trial steering committee is directed by the principal investigator and includes two independent researchers and one statistician, who are not part of the same institution as any of the members of the study team. The trial steering committee is responsible to ensure the protocol is implemented as planned, review study progress on a regular basis, and uphold good clinical practice at all times. The coinvestigators, research assistants, and technicians of the trial will be responsible for all aspects of the logistics and organization of the trial.

### Composition of the data monitoring committee, its role, and reporting structure {21a}

An independent data monitoring committee comprises two independent experts in statistics, who are not part of the same institution as any of the members of the study team. They will be responsible to inspect clinical data collected during the study period, review interim analysis, and report back to the investigators if any action is required.

### Adverse event reporting and harms {22}

Adverse events will be checked by the investigators at every follow-up visit (i.e., every 2 months). Any adverse event, whether they are related to the study or not, will be recorded in the adverse effect report form provided by the Hong Kong Hospital Authority using standard adverse event language. A serious adverse event will be reported to the Hong Kong Hospital Authority Research Ethics Committee within 24 h of the event. The principal investigator will be responsible to follow the management of the serious adverse event until resolution or conclusion. The investigators and the trial steering committee will determine whether an adverse event or serious adverse event is related to the study drug.

### Frequency and plans for auditing trial conduct {23}

A team of clinical research coordinators independent of the investigators will monitor that the data reported in the clinical research forms are complete and accurate, ensure all adverse events and serious adverse events are recorded and reported, and confirm study drugs to be stored and distributed according to the good clinical practice every 3 months.

### Plans for communicating important protocol amendments to relevant parties (e.g., trial participants, ethical committees) {25}

Any amendment to the protocol will be submitted by the principal investigator to be approved by the Hong Kong Hospital Authority Research Ethics Committee and Hong Kong Food and Health Bureau Research Secretariat before implementation.

## Dissemination plans {31a}

Trial results will be presented at international scientific conferences and published in peer-reviewed scientific journals.

## Discussion

Glaucoma neuroprotection trials have been relied on event analysis of VF results for evaluation of treatment outcome, which may take 4 to 5 years to complete [4, 29]. We engineered various designs into the study to enable the detection of a potential treatment effect in 2 years. These include the adoption of (A) a structural (i.e., progressive RNFL thinning) instead of a functional biomarker (e.g., VF progression) as the primary outcome measure, (B) a clustered paradigm to collect multiple measurements at the beginning and at the end of the trial [30], (C) a frequent follow-up schedule at 2-month intervals, and (D) a trend analysis instead of an event analysis to compare the change in RNFL thickness between treatment groups [31], as well as (E) the enrollment of progressing (i.e., eyes with progressive RNFL/GCIPL thinning evident in GPA/TPA) instead of stable glaucoma patients.

Daily NR intake at 300 mg/day is considered to be safe because 300 mg/kg/day represents the no-observed-adverse-effect-level [14]. Whereas many ongoing clinical trials investigate NR at a dosage of ≥1000 mg/day, we prescribe NR at 300 mg/day because oral administration of NR at 300 mg has been shown to attain similar levels of NAD elevation in the blood at 24 h compared with oral administration of NR at 1000 mg in healthy volunteers [6].

Lowering the IOP is currently the only treatment option for glaucoma patients. Our clinical trial has the potential to demonstrate the feasibility of neuroprotection in glaucoma, which is pivotal to prevent or slow glaucoma blindness in patients with progressive loss in vision despite adequate control of IOP.

## Trial status

Study recruitment started on 9 March 2020, and the recruitment completion date is 8 March 2021. Protocol version 5 (8 Nov 2020).

## Abbreviations

EMGT: Early manifest glaucoma trial
FDR: False discovery rate
GCIPL: Ganglion cell inner plexiform layer
GPA: Guided Progression Analysis
IOP: Intraocular pressure
NAD: Nicotinamide adenine dinucleotide
NR: Nicotinamide riboside
OCT: Optical coherence tomography
POAG: Primary open-angle glaucoma
RNFL: Retinal nerve fiber layer
SITA: Swedish interactive thresholding algorithm
TPA: Trend-based Progression Analysis
VF: Visual field
