# Correction to: Nicotinamide riboside as a neuroprotective therapy for glaucoma: study protocol for a randomized, double-blind, placebo-control trial

**DOI:** 10.1186/s13063-022-06079-1

**Published:** 2022-02-10

**Authors:** Christopher Kai Shun Leung, Seraph Tianmin Ren, Poemen Pui Man Chan, Kelvin Ho Nam Wan, Aziz Ka Wai Kam, Gilda Wing Ki Lai, Vivian Sheung Man Chiu, Match Wai Lun Ko, Cedric Ka Fai Yiu, Marco Chak Yan Yu

**Affiliations:** 1 Department of Ophthalmology, LKS Faculty of Medicine, The University of Hong Kong, Hong Kong, People’s Republic of China; 2 Hong Kong Eye Hospital, Hong Kong, People’s Republic of China; 3 Department of Ophthalmology and Visual Sciences, The Chinese University of Hong Kong, Hong Kong, People’s Republic of China; 4 Department of Mechanical Engineering, University of Hong Kong, Hong Kong, People’s Republic of China; 5 Department of Applied Mathematics, Hong Kong Polytechnic University, Hong Kong, People’s Republic of China; 6 Singapore Eye Research Institute, Singapore, Singapore

**Correction to: Leung 23:45 (2022)**

**https://doi.org/10.1186/s13063-021-05968-1**

Following the publication of the original article [1], we were notified that the received date of the paper was 26 November 2020, and not 8 March 2021.

The original article has been corrected.

## References

[1] Leung (2022). Nicotinamide riboside as a neuroprotective therapy for glaucoma: study protocol for a randomized, double-blind, placebo-control. Trial.

